# Automatic quantification of left ventricular function by medical students using ultrasound

**DOI:** 10.1186/s12880-020-00430-1

**Published:** 2020-03-16

**Authors:** Jahn Frederik Grue, Sigurd Storve, Håvard Dalen, Ole Christian Mjølstad, Stein O. Samstad, Torfinn Eriksen-Volnes, Hans Torp, Bjørn Olav Haugen

**Affiliations:** 1 Department of Circulation and Medical Imaging, Faculty of Medicine and Health Sciences, Norwegian University of Science and Technology, NTNU, Prinsesse Kristinas gate 3, 7030 Trondheim, Norway; 2 Clinic of Cardiology, St. Olav′s Hospital, Trondheim University Hospital, Trondheim, Norway; 3 Department of Internal Medicine, Levanger Hospital, Nord-Trøndelag Hospital Trust, Levanger, Norway

**Keywords:** Agreement, Automatic, Longitudinal, Tissue Doppler

## Abstract

**Background:**

Automatic analyses of echocardiograms may support inexperienced users in quantifying left ventricular (LV) function. We have developed an algorithm for fully automatic measurements of mitral annular plane systolic excursion (MAPSE) and mitral annular systolic (S′) and early diastolic (e′) peak velocities. We aimed to study the influence of user experience of automatic measurements of these indices in echocardiographic recordings acquired by medical students and clinicians.

**Methods:**

We included 75 consecutive patients referred for echocardiography at a university hospital. The patients underwent echocardiography by clinicians (cardiologists, cardiology residents and sonographers), who obtained manual reference measurements of MAPSE by M-mode and of S′ and e′ by colour tissue Doppler imaging (cTDI). Immediately after, each patient was examined by 1 of 39 medical students who were instructed in image acquisition on the day of participation. Each student acquired cTDI recordings from 1 to 4 patients. All cTDI recordings by students and clinicians were analysed for MAPSE, S′ and e′ using a fully automatic algorithm. The automatic measurements were compared to the manual reference measurements.

**Results:**

Correct tracking of the mitral annulus was feasible in 50 (67%) and 63 (84%) of the students’ and clinicians’ recordings, respectively (*p* = 0.007). Image quality was highest in the clinicians’ recordings. Mean difference ± standard deviation of the automatic measurements of the students’ recordings compared to the manual reference was − 0.0 ± 2.0 mm for MAPSE, 0.3 ± 1.1 cm/s for S′ and 0.6 ± 1.4 cm/s for e′. The corresponding intraclass correlation coefficients for MAPSE, S′ and e′ were 0.85 (good), 0.89 (good) and 0.92 (excellent), respectively. Automatic measurements from the students’ and clinicians’ recordings were in similar agreement with the reference when mitral annular tracking was correct.

**Conclusions:**

In case of correct tracking of the mitral annulus, the agreement with reference for the automatic measurements was overall good. Low image quality reduced feasibility. Adequate image acquisition is essential for automatic analyses of LV function indices, and thus, appropriate education of the operators is mandatory. Automatic measurements may help inexperienced users of ultrasound, but do not remove the need for dedicated education and training.

## Background

Automatic quantification of left ventricular (LV) function from echocardiographic recordings could assist inexperienced clinicians who perform focused cardiac ultrasound and improve workflow for cardiologists. We have developed an algorithm for fully automatic measurements of mitral annular plane systolic excursion (MAPSE), mitral annular systolic peak velocity (S′) and mitral annular early diastolic peak velocity (e′) [1, 2], all of which are echocardiographic indices of LV longitudinal function and have prognostic value in heart failure [3–6]. The algorithm currently operates on recordings from high-end scanners. It can be implemented in future hand-held ultrasound devices, which as of today offer few options for quantitative assessment of LV function.

In previous studies, we found that the automatic measurements were in good agreement with manual measurements by experienced cardiologists [7] and that the automatically measured indices could be used to detect LV dysfunction [8] when the algorithm analysed ultrasound recordings by experts. In the present study, we aimed to evaluate the influence of users’ experience in echocardiography on the feasibility and accuracy of the automatic measurements, by comparing automatic measurements in recordings by medical students and clinicians utilizing manual measurements by clinicians as reference.

## Methods

### Study population

The patient population consisted of inpatients and outpatients referred for echocardiography at the Clinic of Cardiology, St. Olav’s University Hospital, Trondheim, Norway. The inclusion criteria were age ≥ 18 years and referral for echocardiography at the hospital. The exclusion criterion was too low image quality for manual interpretation. Patients’ demographics and cardiovascular medical history were obtained from the hospital’s patient charts and the echocardiographic archive.

Medical students from the Norwegian University of Science and Technology (NTNU), Trondheim, Norway, were invited to participate in the study. Invitations were sent through the study program’s official e-mail lists and given at lectures. The students could participate if they had been taught at least cardiac physiology, cardiac anatomy and basic ultrasound imaging (theoretical and/or practical) during their medical studies. The majority of first-year medical students at NTNU participate in a two-hour session where they practise ultrasound imaging supervised by cardiologists.

Written informed consent was obtained from all patients and students. The Norwegian Social Science Data Service and the Clinic of Cardiology Institutional Board approved the study, which was conducted according to the Declaration of Helsinki.

### Echocardiographic examinations

The reference echocardiographic examinations were performed by 5 cardiologists, 3 cardiology residents and 3 sonographers who used a commercially available Vivid E9 scanner with a phased-array M5S-D cardiac transducer (bandwidth 1.5–4.6 MHz) or a Vivid S6 scanner with a phased-array M3S cardiac transducer (bandwidth 1.5–4.0 MHz) (both GE Vingmed Ultrasound, Horten, Norway). The cardiologists interpreted their own recordings. Analyses performed by residents and sonographers were secondly approved or adjusted by a cardiologist. The patients were examined in the left lateral decubitus position. Manual analyses of the reference examinations were performed online, or offline in EchoPAC SWO (version 113, GE Vingmed Ultrasound, Horten, Norway).

On the day of participation, each medical student was given a fifteen-minute explanation of how to acquire an apical 4-chamber view and handed printed image examples of correct and wrong views (Additional Figure 1). Immediately after the echocardiographic examination by one of the clinicians, patients were transferred to another room in the same echocardiographic laboratory for examination performed by a medical student. The students used a Vivid 7 scanner with an M3S cardiac transducer (GE Vingmed Ultrasound, Horten, Norway). Colour tissue Doppler imaging (cTDI) with greyscale visualization was activated. Image sector depth, width and gain settings were operated by the instructor, who had no role in the reference examinations. Students were offered a maximum of 15 min of scanning per patient. The students made the decision regarding when to save the recordings, based on when they judged the apical 4-chamber view to be of decent quality. The students were then told to keep the transducer in a steady position for 15 s, and the instructor saved the recording. If the student had time left and wanted to try again, the procedure was repeated. Only the last recording was included in the analyses. The number of attempts was not counted, but all students finished within the assigned time of 15 min.

### Manual reference measurements

The clinicians obtained the manual reference measurements from apical 4-chamber recordings online or in EchoPAC SWO. MAPSE was measured by conventional or reconstructed M-mode from the septal and lateral side of the mitral annulus. Septal and lateral measurements based on three consecutive cardiac cycles were averaged to calculate MAPSE. Reference measurements of S′ and e′ were obtained from apical 4-chamber cTDI recordings using the Q-analysis package in EchoPAC SWO. A 5 × 5 mm circular region of interest was positioned in the septal and lateral LV wall adjacent to the mitral annulus. Septal and lateral measurements based on three consecutive cardiac cycles were averaged.

### Automatic measurements

**Fig. 1:**
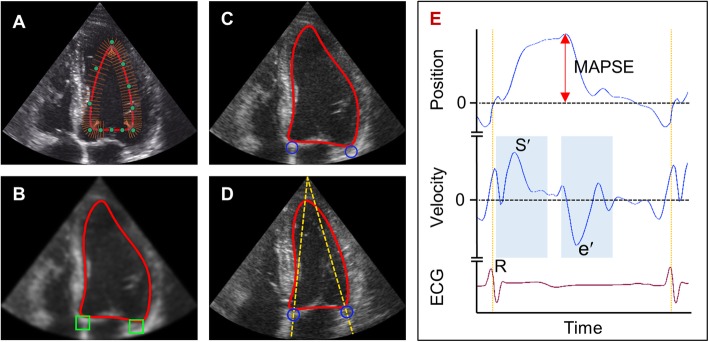
Acquisition of automatic measurements from apical 4-chamber colour tissue Doppler imaging recordings. **a** A deformable model is fitted to the left ventricle by edge-detection on B-mode frames. **b** Search for the mitral annulus. The brightest pixel within each green box is assumed to be from the annulus. **c** and **d** The positions of the tracked points (blue circles) are updated by trapezoidal integration of the Doppler velocity signal, and move straight towards the probe origin (yellow, dashed lines). **e** Position- and velocity data from the tracking points are analysed. The mitral annular plane systolic excursion (MAPSE) is estimated by finding the difference between the start and peak position. The mitral annular peak velocities (S′ and e′) are found within pre-defined time intervals (light blue regions)

The algorithm for the automatic measurements has been thoroughly described previously [7]. Figure 1 illustrates the measurement acquisition. All measurements were calculated by averaging septal and lateral measurements from three cardiac cycles. The apical 4-chamber cTDI recordings acquired by the students and the clinicians were transferred to a laptop computer, converted from DICOM (Digital Information and Communication in Medicine) to in-house file format and analysed using the algorithm. The algorithm was designed to fit a deformable model to the left ventricle and to track the mitral annulus without any human influence. The tracking of the mitral annulus was assessed visually. The tracking was defined as correct if the septal and lateral points of the model both tracked the mitral annulus for three consecutive cardiac cycles. The mean computation time per automatic measurement acquisition was 1.1 s. The analyses of automatic measurements on clinicians’ recordings were done to evaluate how level of ultrasound experience affected the measurements.

### Assessment of image quality

To evaluate the image quality of the apical 4-chamber cTDI recordings by the medical students and clinicians, the following image quality indices were noted: The offset of the apex relative to the image centre, the angle of the LV long-axis, number of visual endocardial segments (0 to 6), presence of significant reverberations, and eventual type of image projection. All image quality indices were measured offline (EchoPAC SWO, version 201) at end-diastole in the last cardiac cycle of each recording. Additional Figure 2 illustrates the image quality assessment.

### Comparisons of automatic measurements and manual reference measurements

In four separate analyses, automatic measurements from student recordings (*Auto Student*) and clinician recordings (*Auto Clinician*) were compared to the reference: 1) All student recordings (*Auto Student*_all_), 2) student recordings where the algorithm correctly tracked the mitral annulus (*Auto Student*_correct_), 3) all clinician recordings (*Auto Clinician*_all_) and 4) clinician recordings where the algorithm correctly tracked the mitral annulus (*Auto Clinician*_correct_).

### Statistics

All statistical analyses were done in SPSS (version 25, IBM Corp, Armonk, NY, USA). Distributions of continuous variables were checked in histograms. Continuous variables are reported as mean ± standard deviation (SD) or as median (1st – 3rd quartile), unless noted otherwise. Categorical variables are reported as number of cases (%). Sample size was based on experience from previous studies by our research group [9, 10].

The number of student and clinician recordings with correct tracking of the mitral annulus was compared with a two-tailed McNemar test, where a *p*-value < 0.05 was considered statistically significant.

To evaluate agreement between automatic and reference measurements, the mean difference, 95% limits of agreement, mean error and intraclass correlation coefficient (ICC) were used as indicators, and Bland-Altman plots [11] were made. Differences were computed as automatic minus reference measurements. The 95% limits of agreement were calculated as the mean difference ± 1.96 SD. The mean error was calculated as the ratio of the absolute difference between measurements to the mean of both measurements, in percent. The ICC was calculated by a mean-rating (k = 2), absolute-agreement, two-way random-effects model (ICC (2,2)) [12]. The intraclass correlation was classified as poor (< 0.50), moderate (0.50–0.74), good (0.75–0.89) or excellent (≥0.90) [12].

## Results

### Patients and medical students

A total of 80 patients and 39 students participated in the study. The inclusion period was between March and June 2015. No patients were excluded due to low image quality. Three examinations had corrupt student cTDI recordings. Two of the reference examinations were missing cTDI. Thus, recordings from 75 patients by 39 medical students and 11 clinicians were included in the analyses. The median study year for the students was 3 (1–4) of 6. The students examined 3 (1–4) patients each, and the 11 clinicians examined 3 (2–8) patients each. The patient population was heterogeneous (50 (67%) men, mean age 64 ± 14 years) with several cardiac conditions (Table 1). Left ventricular function ranged from normal to severely impaired (Table 2).

**Table 1:** Demographics and morbidity of the 75 patients

Age [years]	64 ± 14
Male gender	50 (67%)
Body mass index [kg/m^2^]	27.2 ± 5.2
Body surface area [m^2^]	2.0 ± 0.2
Diabetes	13 (17%)
Hypertension	40 (53%)
Coronary artery disease	25 (33%)
Cardiac surgery	12 (16%)
Current severe valvular disease	8 (11%)
Current atrial fibrillation	8 (11%)

Data are presented as mean ± standard deviation or number of patients (%)

**Table 2:** Elected echocardiographic findings in the 75 patients

	Mean ± SD	Range (min – max)
Ejection fraction [%]	52 ± 9	28–70
IVSd [cm]	1.1 ± 0.2	0.6–1.8
LVIDd [cm]	5.0 ± 0.8	3.4–7.0
LVPWd [cm]	1.0 ± 0.2	0.6–1.5
E/A	1.1 ± 0.5	0.5–2.6
E deceleration time [ms]	205 ± 68	91–408
E/e′ (average of septal and lateral, pwTDI)	10.9 ± 5.3	3.5–27.0
S/D	1.2 ± 0.3	0.6–2.0
TR velocity [m/s]	2.5 ± 0.4	2.0–3.8

*E/A* Ratio of early to late diastolic mitral velocity, *E/e′* Ratio of E to mitral annular early diastolic peak velocity, *IVSd* Interventricular septum end-diastolic thickness, *LVIDd* Left ventricular internal end-diastolic diameter, *LVPWd* Left ventricular posterior wall end-diastolic thickness, *max* Maximum, *min* Minimum, *pwTDI* Pulsed wave tissue Doppler imaging, *S/D* Ratio of systolic to diastolic pulmonary vein velocity, *SD* Standard deviation, *TR* Tricuspid regurgitation

### Mitral annular tracking and image quality

In the 75 student recordings, the algorithm tracked the mitral annulus correctly in 50 (67%) of the cases, while on the 75 clinician recordings, the tracking was correct in 63 (84%) cases (*p* = 0.007 for difference). There were 4 (5%) cases where the tracking of the mitral annulus was correct on the student’s recording, but incorrect on the clinician’s recording. The tracking results were similar in 54 (72%) of all 75 patients. Examples of failed tracking are shown in Additional Figure 3. Student recordings, especially those with incorrect tracking, were of lower quality than those by clinicians, with higher misalignment of the left ventricle and more reverberation artifacts (Table 3).

**Table 3:** Characteristics of recordings with correct and wrong mitral annular tracking

	Recordings by students		Recordings by clinicians	
	Correct tracking (*n* = 50)	Wrong tracking (*n* = 25)	Correct tracking (*n* = 63)	Wrong tracking (*n* = 12)
Visible endocardial segments	4 (3–5)	3 (2–4)	5 (4–6)	5 (4–6)
Apex offset [mm]	5 (2–9)	7 (4–21)	4 (2–6)	5 (2–6)
LV long-axis angle [°]	9 (4–12)	14 (8–17)	4 (3–7)	3 (0–7)
Non-A4C views	9 (18%)	13 (52%)	0	0
Reverberations	16 (32%)	13 (52%)	14 (22%)	9 (75%)

Data are presented as median (1st – 3rd quartile) or number of cases (%). *A4C* Apical 4-chamber, *LV* Left ventricular

### Automatic measurements compared to manual reference measurements

The agreement between *Auto Student*_all_ and the manual reference, expressed as mean difference ± SD, was − 0.8 ± 3.6 mm for MAPSE, 0.1 ± 1.8 cm/s for S′ and 0.3 ± 2.1 cm/s for e′. The corresponding intraclass correlation was moderate for MAPSE and S′, and good for e′, with ICC (95% CI) of 0.63 (0.42–0.76), 0.74 (0.58–0.83) and 0.82 (0.72–0.89), respectively. The agreement improved by excluding cases where the mitral annular tracking was incorrect. *Auto Student*_correct_ and the manual reference had a mean difference ± SD of − 0.0 ± 2.0 mm for MAPSE, 0.3 ± 1.1 cm/s for S′ and 0.6 ± 1.4 cm/s for e′. The corresponding intraclass correlation was good for MAPSE and S′, and excellent for e′, with ICC (95% CI) of 0.85 (0.74–0.92), 0.89 (0.81–0.94) and 0.92 (0.83–0.96), respectively.

Between *Auto Clinician*_all_ and the manual reference, the mean difference ± SD was − 0.8 ± 2.8 mm for MAPSE, 0.1 ± 1.3 cm/s for S′ and 0.2 ± 1.4 cm/s for e′. The corresponding intraclass correlation was moderate for MAPSE, good for S′, and excellent for e′, with ICC (95% CI) of 0.70 (0.53–0.81), 0.86 (0.78–0.91) and 0.93 (0.89–0.96), respectively. The highest agreement was between *Auto Clinician*_correct_ and the manual reference, where the mean difference ± SD was − 0.1 ± 2.0 mm for MAPSE, 0.4 ± 0.7 cm/s for S′, and 0.4 ± 1.1 cm/s for e′. The corresponding intraclass correlation was good for MAPSE, and excellent for S′ and e′, with ICC (95% CI) of 0.82 (0.71–0.89), 0.94 (0.86–0.97) and 0.94 (0.90–0.97), respectively.

**Fig. 2:**
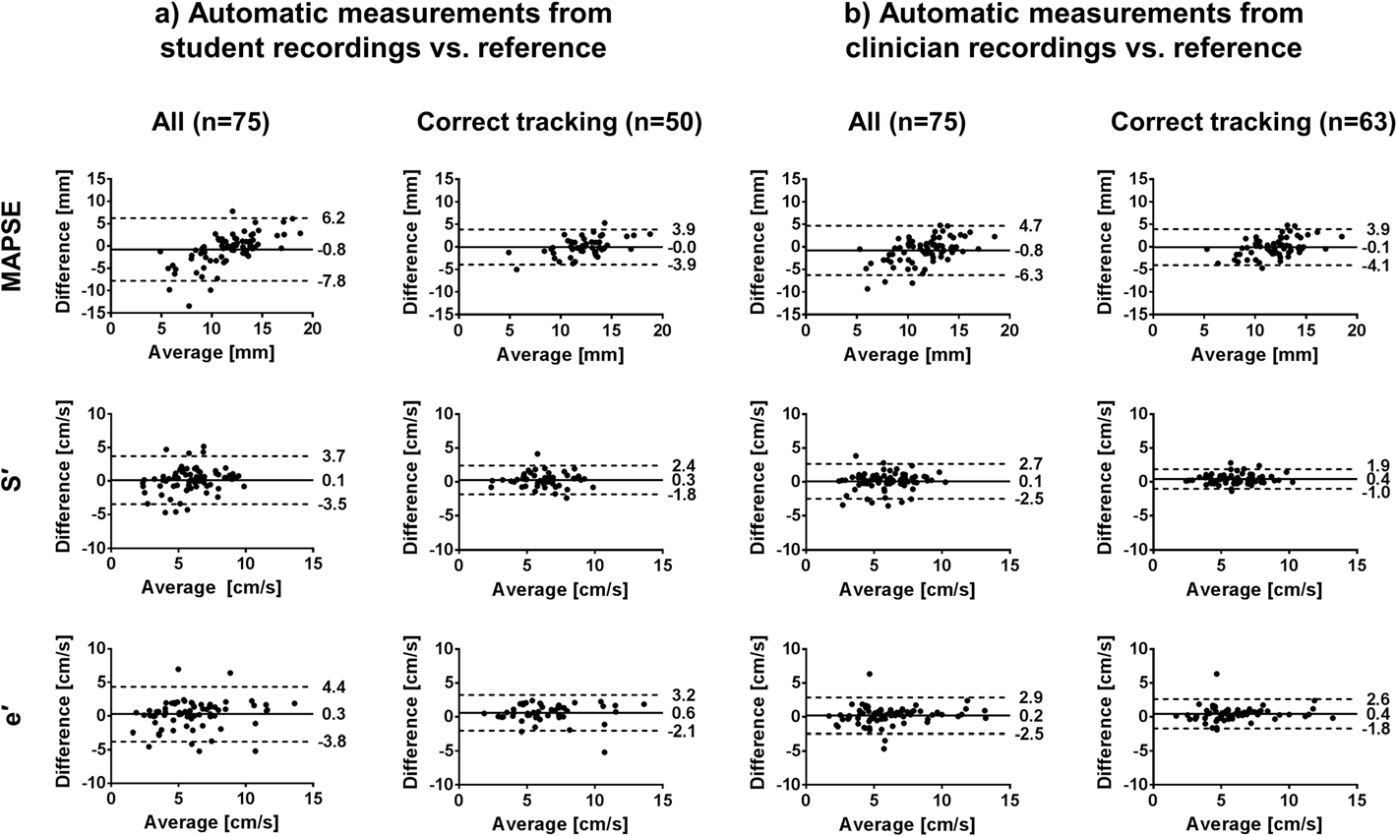
The agreement between automatic and reference measurements. Each dot represents measurements from one patient. The difference is calculated as automatic minus reference measurements. Solid, horizontal lines show the mean differences. Dashed, horizontal lines show the 95% limits of agreement. e′, mitral annular early diastolic peak velocity; MAPSE, mitral annular plane systolic excursion; S′, mitral annular systolic peak velocity

The Bland-Altman plots in Fig. 2 show the agreement between the automatic measurements and the manual reference measurements. Additional Table 1 shows all estimates of agreement with 95% confidence intervals.

## Discussion

In the present study, we found that automatic measurements of mitral annular motion indices derived from echocardiographic recordings acquired by medical students were reasonably similar to manual reference measurements by clinicians in the majority of cases. However, a substantial part of the student recordings were of low quality, which reduced feasibility. Competence in obtaining an apical 4-chamber view is essential for valid automatic measurements.

To the best of our knowledge, no other research group has evaluated automatic measurements of echocardiographic indices from recordings by inexperienced users. In a study by Dhutia and colleagues it was shown that S′ and e′ could be measured automatically from pulsed wave tissue Doppler spectra [13]. The agreement with the manual reference was as good as the interobserver agreement between three human observers. Their results are comparable to what we found for *Auto Clinician*_correct_. In our study, the agreement with the reference for the automatic measurements of MAPSE was close to what has been reported for interobserver agreement among experts using M-mode [14]. For all mitral annular motion indices, the Bland-Altman plots revealed that most of the severely underestimated automatic measurements (located in the lower left part among the scatters in Fig. 2) were removed by excluding cases of incorrect mitral annular tracking. Thus, we argue that the automatic algorithm can measure the mitral annular motion indices adequately as long as the mitral annular tracking is correct.

As mentioned, examinations using hand-held ultrasound devices are generally interpreted visually [15]. The use of quantitative measures may improve the diagnostic yield and is recommended [16]. Others have shown that non-specialists including medical students and resident physicians can evaluate systolic function by interpreting ultrasound images after dedicated training [17–19]. As diastolic function cannot be evaluated simply by studying two-dimensional (2D) images, it is especially quantitative measurements of e′, an important index of diastolic function [20], that may assist inexperienced users the most among the indices we measured in this study. As the present study did not compare visual and quantitative assessment of LV function, it is uncertain whether the automatic measurements can outperform visual assessment.

We presumed that the apical 4-chamber view is a relatively easy view to obtain compared to other cardiac views. Importantly, it is clear that substantially more training than provided in this study is needed before performing diagnostic ultrasound. However, diagnostics was not the purpose of the study, and we do not recommend performing diagnostic ultrasound with such limited training. In line with the European Association of Cardiovascular Imaging’s (EACVI) viewpoint on education for focused cardiac ultrasound, the students should have been trained until they had reached an adequate level of skills and were able to acquire the apical 4-chamber view [15, 16]. This may not require much resources in terms of teachers. A recent study showed that lending students hand-held ultrasound devices for self-practice and either providing them with e-learning or a practical course gave them equal abilities in conducting a short, structured ultrasound examination [21]. Hands-on self-training augmented by e-learning could be a preferable approach for teaching in future studies, but this approach was not evaluated in this study. In post-hoc analyses (Additional Table 2), we examined if the tracking results on student recordings were associated with LV function (ejection fraction), age and body mass index (BMI). There were no significant differences, although the patients in whom the tracking failed tended to have higher mean BMI (*p* = 0.07). This is plausible considering that patients with high BMI may be more challenging to examine by ultrasound [9].

There are several limitations to this study. First, the short-term training of the medical students was insufficient. More comprehensive training is recommended for clinical practice. Thus, the clinical benefit cannot be inferred from the results. Second, the students used a Vivid 7 scanner for all recordings, while the clinicians used a newer Vivid E9 with better image processing in 72 (96%) of the cases. As the algorithm is sensitive to reverberations, this might have contributed to lower quality of the automatic measurements from student recordings. Third, only one measurement of each type (manual and automatic) was acquired per patient, so the reliability of each method could not be evaluated. Fourth, a sample size calculation was not conducted a priori. Fifth, the automatic measurements of S′ and e′ from clinicians’ recordings were obtained from the same recordings as the manual reference measurements. Sixth, only indices of LV longitudinal function were measured automatically, and the presented results should not be generalized to other echocardiographic indices. Finally, we did not study the clinical impact of the automatic LV longitudinal motion indices in this study. In a previous study we have shown the diagnostic utility of the method [8]. For diagnostic purposes, high quality recordings and accurate quantification are of utmost importance.

The software for automatic measurements of MAPSE from 2D greyscale images is available as a research app in a commercially available hand-held ultrasound device (Vscan Extend, GE Vingmed Ultrasound, Horten, Norway). The quality and diagnostic utility of this method in the hands of operators with different levels of experience in ultrasound will be evaluated in future studies.

## Conclusions

Automatic measurements of echocardiographic indices of mitral annular motion from recordings by medical students may provide quantitative data comparable to measurements by clinicians in a heterogeneous patient population. Overall, the feasibility of the automatic measurements was not sufficient in the hands of the very inexperienced users. Thus, adequate training of users in image acquisition is mandatory before implementing automatic measurements in clinical practice. Automatic quantitative analyses of LV function indices may help both inexperienced and experienced users if images are properly acquired and failures of the automatic procedure are identified.

## Supplementary information

**Additional file 1: Additional Figure 1.** The printed instructions given to medical students before examining patients.

**Additional file 2: Additional Figure 2.** Assessment of image quality.

**Additional file 3: Additional Figure 3.** Examples of failed tracking of the mitral annulus on student recordings.

**Additional file 4: Additional Table 1.** Agreement between automatic and manual reference measurements.

**Additional file 5: Additional Table 2.** Mitral annular tracking in student recordings and patient characteristics.

## Data Availability

The datasets used and/or analysed during the current study are available from the corresponding author on reasonable request.

## Abbreviations

2D: Two-dimensional
BMI: Body mass index
cTDI: Colour tissue Doppler imaging
DICOM: Digital Information and Communication in Medicine
e′: Mitral annular early diastolic peak velocity
EACVI: European Association of Cardiovascular Imaging
ICC: Intraclass correlation coefficient
LV: Left ventricular
MAPSE: Mitral annular plane systolic excursion
NTNU: Norwegian University of Science and Technology
S′: Mitral annular systolic peak velocity
SD: Standard deviation
