# Supplementary material for: Automatic quantification of left ventricular function by medical students using ultrasound
Source: BMC Med Imaging. 2020 Mar 16;20:29. doi: 10.1186/s12880-020-00430-1 (PMC7077164; doi:10.1186/s12880-020-00430-1)
Supplement: Supplementary file 1 — Additional file 1: Additional Figure 1. The printed instructions given to medical students before examining patients. [file 12880_2020_430_MOESM1_ESM.pdf]

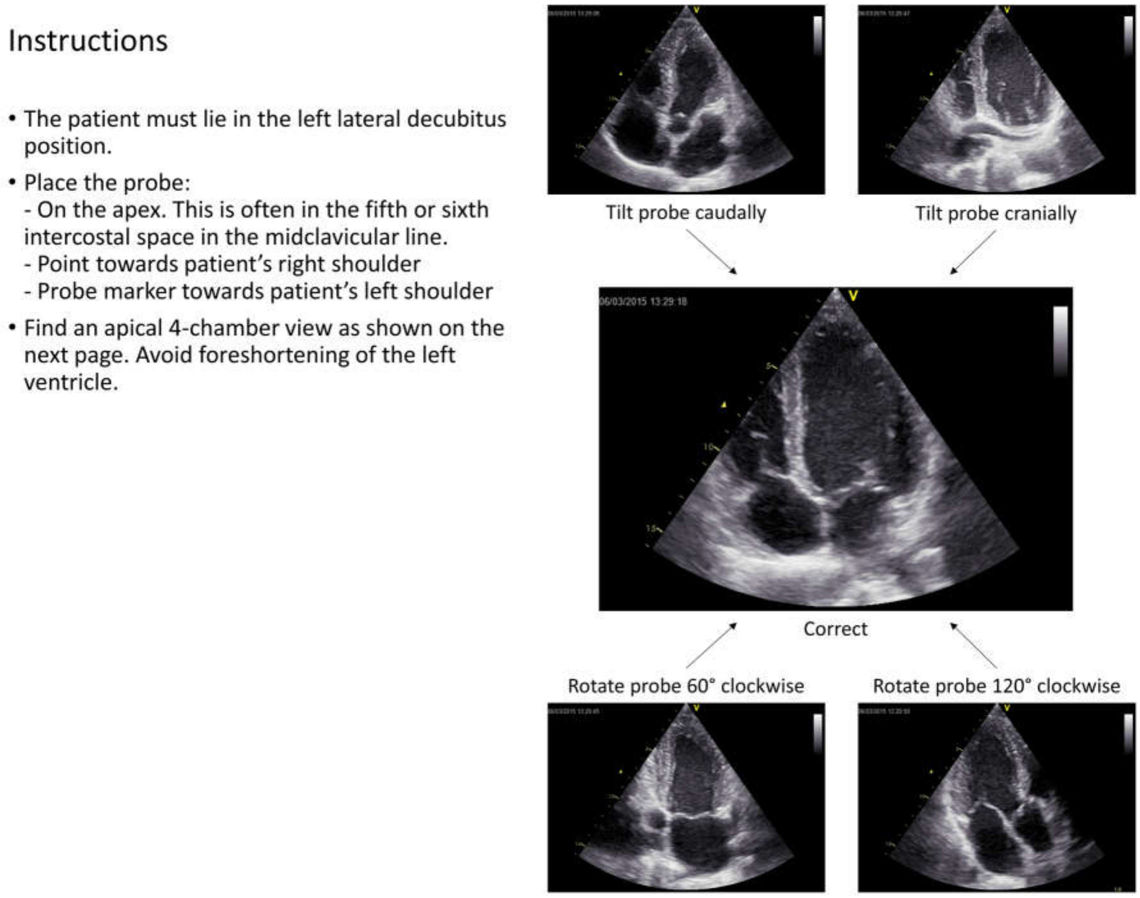

Additional Figure 1. The printed instructions given to medical students before examining patients.
