# Supplementary material for: Automatic quantification of left ventricular function by medical students using ultrasound
Source: BMC Med Imaging. 2020 Mar 16;20:29. doi: 10.1186/s12880-020-00430-1 (PMC7077164; doi:10.1186/s12880-020-00430-1)
Supplement: Supplementary file 2 — Additional file 2: Additional Figure 2. Assessment of image quality. [file 12880_2020_430_MOESM2_ESM.pdf]

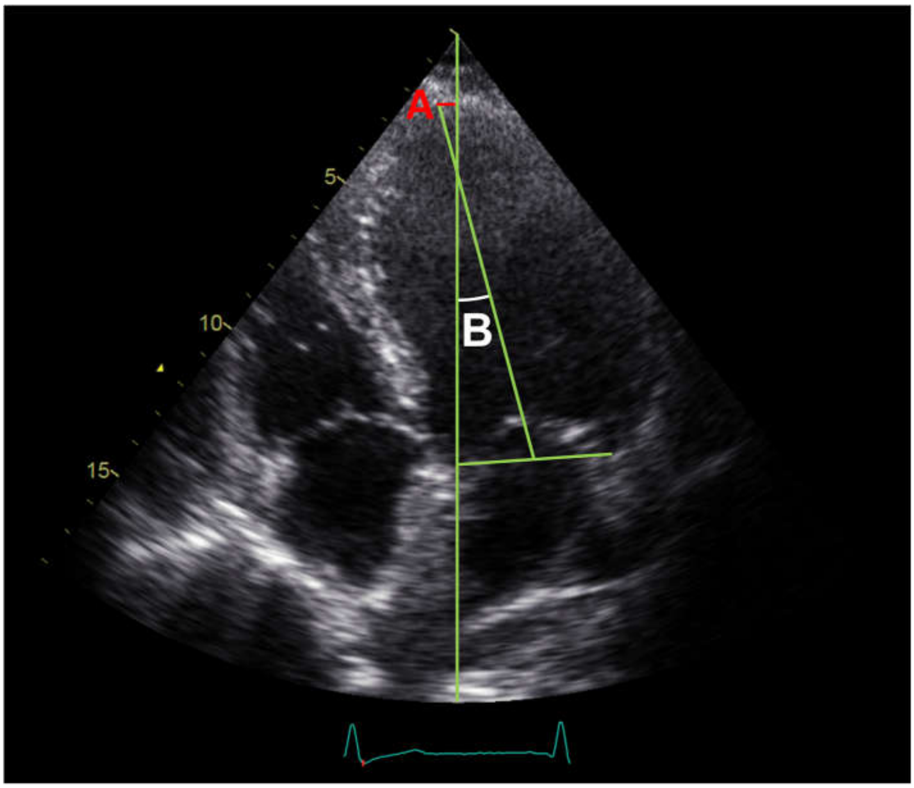

Additional Figure 2. Assessment of image quality. Apical 4-chamber view recorded by a medical student. 4 of 6 endocardial segments of the left ventricle are visible. The apex is offset 5 mm from the centre (A), and the left ventricular long-axis angle is 15 degrees (B).
