# Supplementary material for: Automatic quantification of left ventricular function by medical students using ultrasound
Source: BMC Med Imaging. 2020 Mar 16;20:29. doi: 10.1186/s12880-020-00430-1 (PMC7077164; doi:10.1186/s12880-020-00430-1)
Supplement: Supplementary file 3 — Additional file 3: Additional Figure 3. Examples of failed tracking of the mitral annulus on student recordings. [file 12880_2020_430_MOESM3_ESM.pdf]

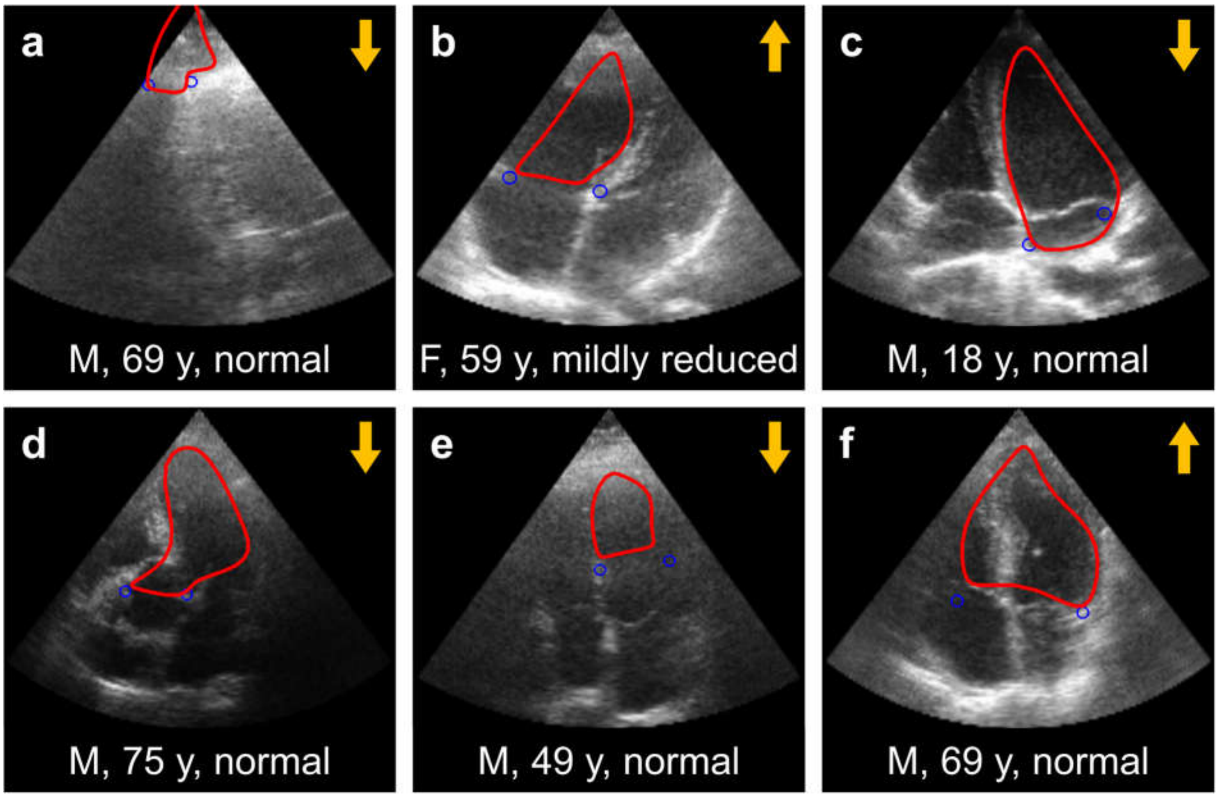

Additional Figure 3. Examples of failed tracking of the mitral annulus on student recordings. The patients' gender (F, female; M, male), age (y, years) and left ventricular function is indicated. The red line indicates the deformable model's attempt in segmentation of the left ventricle. The two blue rings are the tracking points. Arrows pointing upwards and downwards indicate over- and underestimation of mitral annular motion indices. a) All wrong projection resulting in tracking points outside of the heart. b) Oblique projection due to medial placement of the probe, leading to segmentation of the right ventricle and tracking of the tricuspid annular plane. c) Foreshortened view due to caudal angulation of the probe, causing tracking of the left atrial posterior wall instead of the mitral annulus. d) Apical five chamber view due to cranial angulation of the probe, causing tracking of the aorta. e) Reverberation noise through the middle of the left ventricle in an acceptable projection, making the tracking points stick to the reverberation. f) Failed tracking in a recording of acceptable quality, where the septal point tracks the septal tricuspid leaflet instead of the mitral annulus.
