# Supplementary material for: Automatic quantification of left ventricular function by medical students using ultrasound
Source: BMC Med Imaging. 2020 Mar 16;20:29. doi: 10.1186/s12880-020-00430-1 (PMC7077164; doi:10.1186/s12880-020-00430-1)
Supplement: Supplementary file 4 — Additional file 4: Additional Table 1. Agreement between automatic and manual reference measurements. [file 12880_2020_430_MOESM4_ESM.docx]

| **Additional Table 1. Agreement between automatic and manual reference measurements.** | | | | | | | |
| --- | --- | --- | --- | --- | --- | --- | --- |
| MAPSE | n | *Auto* mean ± SD | *Ref* mean ± SD | Mean difference (95% CI) | 95% LoA (95% CI) | Mean error (95% CI) | ICC (95% CI) |
| *Auto Student*_all_ vs. *Ref* | 75 | 11.3 ± 4.3 mm | 12.1 ± 2.2 mm | -0.8 (-1.6 ‒ 0.0) mm | -7.8 ‒ 6.2 (-8.5 ‒ 6.9) mm | 26.2 (18.5 ‒ 34.0) % | 0.63 (0.42 ‒ 0.76) |
| *Auto Clinician*_all_ vs. *Ref* | 75 | 11.3 ± 3.6 mm | 12.1 ± 2.2 mm | -0.8 (-1.4 ‒ -0.2) mm | -6.3 ‒ 4.7 (-6.8 ‒ 5.2) mm | 21.0 (15.2 ‒ 26.7) % | 0.70 (0.53 ‒ 0.81) |
| *Auto Student*_correct_ vs. *Ref* | 50 | 12.3 ± 3.2 mm | 12.3 ± 2.3 mm | -0.0 (-0.6 ‒ 0.5) mm | -3.9 ‒ 3.9 (-4.4 ‒ 4.3) mm | 13.4 (9.4 ‒ 17.5) % | 0.85 (0.74 ‒ 0.92) |
| *Auto Clinican*_correct_ vs. *Ref* | 63 | 11.9 ± 3.0 mm | 12.0 ± 2.2 mm | -0.1 (-0.6 ‒ 0.4) mm | -4.1 ‒ 3.9 (-4.5 ‒ 4.4) mm | 14.0 (10.9 ‒ 17.1) % | 0.82 (0.71 ‒ 0.89) |
| S′ |  |  |  |  |  |  |  |
| *Auto Student*_all_ vs. *Ref* | 75 | 6.0 ± 2.2 cm/s | 5.8 ± 1.8 cm/s | 0.1 (-0.3 ‒ 0.5) cm/s | -3.5 ‒ 3.7 (-3.8 ‒ 4.1) cm/s | 24.9 (18.4 ‒ 31.4) % | 0.74 (0.58 ‒ 0.83) |
| *Auto Clinician*_all_ vs. *Ref* | 75 | 5.9 ± 1.9 cm/s | 5.8 ± 1.8 cm/s | 0.1 (-0.2 ‒ 0.4) cm/s | -2.5 ‒ 2.7 (-2.8 ‒ 2.9) cm/s | 17.7 (12.6 ‒ 22.9) % | 0.86 (0.78 ‒ 0.91) |
| *Auto Student*_correct_ vs. *Ref* | 50 | 6.3 ± 1.8 cm/s | 6.0 ± 1.8 cm/s | 0.3 (-0.0 ‒ 0.6) cm/s | -1.8 ‒ 2.4 (-2.1 ‒ 2.7) cm/s | 13.5 (10.0 ‒ 17.1) % | 0.89 (0.81 ‒ 0.94) |
| *Auto Clinican*_correct_ vs. *Ref* | 63 | 6.2 ± 1.8 cm/s | 5.7 ± 1.7 cm/s | 0.4 (0.2 ‒ 0.6) cm/s | -1.0 ‒ 1.9 (-1.2 ‒ 2.0) cm/s | 10.7 (8.3 ‒ 13.1) % | 0.94 (0.86 ‒ 0.97) |
| e′ |  |  |  |  |  |  |  |
| *Auto Student*_all_ vs. *Ref* | 75 | 6.2 ± 2.8 cm/s | 5.9 ± 2.5 cm/s | 0.3 (-0.2 ‒ 0.8) cm/s | -3.8 ‒ 4.4 (-4.2 ‒ 4.8) cm/s | 28.2 (20.9 ‒ 35.6) % | 0.82 (0.72 ‒ 0.89) |
| *Auto Clinician*_all_ vs. *Ref* | 75 | 6.1 ± 2.7 cm/s | 5.9 ± 2.5 cm/s | 0.2 (-0.1 ‒ 0.5) cm/s | -2.5 ‒ 2.9 (-2.7 ‒ 3.1) cm/s | 18.2 (13.3 ‒ 23.1) % | 0.93 (0.89 ‒ 0.96) |
| *Auto Student*_correct_ vs. *Ref* | 50 | 6.7 ± 2.6 cm/s | 6.2 ± 2.6 cm/s | 0.6 (0.2 ‒ 1.0) cm/s | -2.1 ‒ 3.2 (-2.4 ‒ 3.6) cm/s | 17.5 (13.7 ‒ 21.3) % | 0.92 (0.83 ‒ 0.96) |
| *Auto Clinican*_correct_ vs. *Ref* | 63 | 6.2 ± 2.6 cm/s | 5.7 ± 2.5 cm/s | 0.4 (0.2 ‒ 0.7) cm/s | -1.8 ‒ 2.6 (-2.0 ‒ 2.9) cm/s | 15.0 (10.2 ‒ 19.7) % | 0.94 (0.90 ‒ 0.97) |
| *Auto*, automatic measurements; *Auto Clinician*_all_, automatic measurements from all clinician recordings; *Auto Clinician*_correct_, automatic measurements from clinician recordings with correct tracking of the mitral annulus; *Auto Student*_all_, automatic measurements from all student recordings; *Auto Student*_correct_, automatic measurements from student recordings with correct tracking of the mitral annulus; CI, confidence interval; e′, mitral annular early diastolic peak velocity; ICC, intraclass correlation coefficient; LoA, limits of agreement; MAPSE, mitral annular plane systolic excursion; n, number of cases;  *Ref*, reference measurements from clinician recordings; S′, mitral annular systolic peak velocity; SD, standard deviation. | | | | | | | |
