# Supplementary material for: Automatic quantification of left ventricular function by medical students using ultrasound
Source: BMC Med Imaging. 2020 Mar 16;20:29. doi: 10.1186/s12880-020-00430-1 (PMC7077164; doi:10.1186/s12880-020-00430-1)
Supplement: Supplementary file 5 — Additional file 5: Additional Table 2. Mitral annular tracking in student recordings and patient characteristics. [file 12880_2020_430_MOESM5_ESM.docx]

| **Additional Table 2. Mitral annular tracking in student recordings and patient characteristics.** | | | |
| --- | --- | --- | --- |
|  | Correct tracking | Failed tracking | p |
| EF [%] | 52.5 ± 9.3 | 50.4 ± 0.6 | 0.93 |
| Age [years] | 63.5 ± 12.9 | 63.8 ± 17.2 | 0.36 |
| BMI [kg/m^2^] | 26.3 ± 4.0 | 28.7 ± 6.8 | 0.07 |
| Data are mean ± standard deviation. Two-tailed independent t-tests were used. EF, ejection fraction; BMI, body mass index. | | | |
